# *Rickettsia typhi* in Southern California: A Growing Flea-Borne Threat

**DOI:** 10.4269/ajtmh.23-0742

**Published:** 2023-12-18

**Authors:** Lucas S. Blanton

**Affiliations:** Department of Internal Medicine, Division of Infectious Diseases, University of Texas Medical Branch, Galveston, Texas

*Rickettsia typhi* is a flea-borne bacterium that causes an acute undifferentiated febrile illness in humans.^1^ Disease attributed to *R. typhi* has various descriptive names that characterize its ecology and epidemiology. Flea-borne typhus names its vector; murine typhus describes its murid reservoir; and endemic typhus differentiates it from epidemic louse-borne typhus, a more severe illness caused by *Rickettsia prowazekii*.^1^^,^^2^ Classically maintained by rats (*Rattus rattus* and *R. norvegicus*),^2^ *R. typhi* likely also utilizes opossums (*Didelphis virginiana*) as an amplifying host in North America.^3^^–^^6^ Clinically, flea-borne typhus is characterized by fever, headache, malaise, and myalgias. Although rash is often considered characteristic of a rickettsiosis, it occurs in only half of cases. Laboratory abnormalities such as elevated hepatic transaminases, thrombocytopenia, and hyponatremia are often noted.^7^ None of these features is specific to any particular infectious etiology. Thus, flea-borne typhus is easily mistaken for diseases caused by various other pathogens.^8^ The lack of an accurate, rapid diagnostic test employable during the early phase of illness and reliance on serology for diagnosis (antibodies are seldom detected in the first several days of illness),^9^ further clouds our ability to recognize flea-borne typhus and establish its contribution to illness within a community. Despite the difficulties in establishing a diagnosis, it has become clear that flea-borne typhus is becoming increasingly prevalent.^10^^,^^11^

The article published by Yomogida and colleagues^12^ in this issue of the *AJTMH* titled “Surveillance of Flea-borne Typhus in California, 2011–2019” is an important and timely contribution that puts a spotlight on this emerging infectious threat. In California, flea-borne typhus is a notifiable disease and is reported to the California Department of Public Health from county or city health departments via electronically submitted case report forms. A total of 881 cases were reported during these 9 years of surveillance, with most occurring in Los Angeles and Orange Counties (78% and 19% of state cases, respectively). Over this period, the number of cases steadily increased. Whereas 47 cases were reported in 2011, there were 164 reported in 2018 and 140 reported in 2019. A separate report involving some of the same public health team members that contributed to this work confirms this upward trend, with 171 cases identified in California in 2022.^11^

It is estimated that fewer than a third of flea-borne typhus cases are ever diagnosed,^13^ suggesting that the number of cases described by this work merely scratches the surface of the true incidence of flea-borne typhus in California. Several aspects of this article support this notion. First, 83% of cases were hospitalized, with a 4-day median length of stay. A small number (3%) required readmission, and the average time from symptom onset to the collection of a positive antibody test was 12.6 days. For a disease that is often described as mild (the case-fatality rate is 0.4%),^14^ the high proportion hospitalized and length of stay are staggering. When patients are ill enough to be hospitalized, the search for answers and therapeutics drives diagnostic workups. Hence, the number of reported hospitalizations likely represents a small subset of total cases, for whom supportive diagnostic evidence has been obtained. In many of those hospitalized, flea-borne typhus was likely not considered early during their hospital stays, an unfortunate phenomenon that is not unique to California.^15^

The authors also report that 43% of cases occurred in Caucasians, while 33% were in those of Hispanic or Latino race/ethnicity. When considering the population distribution of Los Angeles and Orange Counties, cases of flea-borne typhus are overrepresented by non-Hispanic whites. The authors acknowledge that socioeconomic disparities (e.g., income, healthcare access, and insurance coverage) may play a role in this discrepancy. Indeed, the inability to seek medical care and the requirement of laboratory diagnostic testing to trigger surveillance mechanisms likely lead to underreporting of flea-borne typhus.

Finally, the article reports differences in the frequency of certain clinical features of flea-borne typhus compared with what has been reported elsewhere. Compared with a systematic review of >2000 patients with flea-bore typhus (data from 33 previously published case series),^7^ gastrointestinal symptoms (nausea/vomiting (51% versus 27%), diarrhea (29% versus 19%), and abdominal pain (32% versus 18%)) were more prominent in this California cohort. The frequency of thrombocytopenia was lower than what has been reported (22% versus 42%). Certainly, the retrospective nature of data collection has its limitations, but these discrepancies and the overwhelming use of serology (including single specimens) to support the diagnosis of flea-borne typhus raise another possible explanation for differences in frequencies of signs and symptoms. It is possible that there is a demonstrable seroprevalence of typhus group antibodies within these Southern California communities that drives misclassification of illnesses as flea-borne typhus when patients present with an alternative illness. Considering the clinical dilemma of evaluating an acute undifferentiated febrile illness, this scenario is not hard to imagine. The potential of significant seroreactivity in the community suggests many more infections than are currently recognized. The higher-than-expected seroreactivity to *R. typhi* within other endemic and previously endemic areas supports this possibility.^16^^,^^17^

The evidence suggesting flea-borne typhus is under-recognized in Southern California has broad implications. Yes, flea-borne typhus has been described as mild, but patients infected with *R. typhi* tend to disagree with this sentiment. The fever can extend over 3 weeks, the headache is often quite severe,^18^^,^^19^ and when ill enough to be hospitalized, 10% of patients have required intensive care, with a case-fatality rate approaching 4%.^20^ Severe manifestations, such as meningoencephalitis,^21^ acute kidney injury,^22^ and respiratory failure^23^ can all complicate the course of illness. The recent report of three flea-borne typhus deaths in Los Angeles reinforces the pathogenic potential of *R. typhi*.^11^ It is unlikely that the organism has evolved to gain virulence in Southern California, as *R. typhi* isolates from geographically distant regions are genetically conserved.^24^ Rather, more severe cases are increasingly likely to be reported as the incidence increases. The article by Yomogida et al.^12^ is a call to attention to clinicians and public health authorities nationwide. With the ability of rats to thrive in the vicinity of humans,^25^ the broad geographic distribution of opossums,^26^ and the ubiquity of fleas,^27^ the resurgence of flea-borne typhus in other communities of California, and in other states, is likely.
